# Associations of Neutrophil-to-Lymphocyte Ratio with Cerebral Small Vessel Disease and Functional Outcome in Acute Ischaemic Stroke Patients

**DOI:** 10.3390/life16020337

**Published:** 2026-02-15

**Authors:** Nipit Tieachanpan, Surat Tanprawate, Atiwat Soontornpun, Chayasak Wantaneeyawong, Chutithep Teekaput, Nopdanai Sirimaharaj, Angkana Nudsasarn, Withawat Vuthiwong, Kitti Thiankhaw

**Affiliations:** 1 Division of Neurology, Department of Internal Medicine, Faculty of Medicine, Chiang Mai University, Chiang Mai 50200, Thailand; marknipit@gmail.com (N.T.); surat.tan@cmu.ac.th (S.T.); atiwat.s@cmu.ac.th (A.S.); chayasak.w@cmu.ac.th (C.W.); chutithep.t@cmu.ac.th (C.T.); nopdanai.s@cmu.ac.th (N.S.); angkana4003@gmail.com (A.N.); 2 The Northern Neuroscience Center, Faculty of Medicine, Chiang Mai University, Chiang Mai 50200, Thailand; 3 Division of Diagnostic and Interventional Neuroradiology, Department of Radiology, Faculty of Medicine, Chiang Mai University, Chiang Mai 50200, Thailand; withawat.vuthiwong@cmu.ac.th

**Keywords:** cerebral small vessel disease, functional outcome, neutrophil–lymphocyte ratio, ischaemic stroke, inflammatory biomarkers

## Abstract

Background: The relationship between inflammatory markers and cerebral small vessel disease (CSVD) in patients with acute ischaemic stroke (AIS) remains unclear. This study aimed to investigate the association between simplified inflammatory biomarkers and neuroimaging markers of CSVD. Methods: This retrospective cohort study included patients with AIS who had symptom onset within 72 h and underwent MRI brain between January 2019 and December 2023. The associations between tertiles (T) of the neutrophil-to-lymphocyte ratio (NLR) and CSVD markers were studied using multinomial logistic regression. Functional outcomes at discharge and 90 days, as measured by the modified Rankin Scale (mRS), were also evaluated. Results: A total of 299 eligible patients were included, with a mean age of 65.7 ± 13.8 years and 55.5% (166/299) were male, and categorised into three tertiles of NLR (T1: 101, T2: 101, T3: 97). Patients with a higher NLR tertile had more admission NIHSS (T3 vs. T1: 3 (2, 5) vs. 2 (1, 3), *p* = 0.005). NLR was associated with an increased risk of ≥5 lobar cerebral microbleeds (CMBs) in an unadjusted model (T3 vs. T1: relative risk ratio (RRR), 5.69 (95% confidence interval (CI) 1.21–26.68); *p* = 0.03); however, this was not significant when adjusted for potential confounders (RRR 3.86; 95% CI 0.79–18.89; *p* = 0.10). No significant associations were observed in the remaining neuroimaging markers of CSVD. Patients in the T2 of NLR had a higher likelihood of achieving an mRS of 0–1 at 90 days (RRR 2.16; 95% CI 1.05–4.44; *p* = 0.04) compared to those in T1. Conclusions: In AIS, admission NLR showed a possible association with higher lobar CMB burden in unadjusted analyses, but this was not robust after adjustment, and no consistent relationships were observed with other CSVD markers. Associations with functional outcomes were not uniform across tertiles, and the apparent benefit in the middle NLR tertile should be interpreted cautiously as a potentially non-linear or chance finding, indicating that NLR is not a reliable independent imaging or prognostic marker in this cohort.

## 1. Introduction

Cerebral small vessel disease (CSVD) is a condition characterised by damage to the small veins, arteries, and capillaries in the brain. It has been linked to the narrowing and hardening of these vessels, which reduces blood flow and impairs oxygen exchange [1]. Over the years, CSVD has been recognised as an important risk factor for doubling stroke likelihood [2] and is also associated with long-term neurologic complications, including dementia [3] and changes in mood and personality [4]. Diagnosis still relies heavily on expert neuroimaging interpretation, especially magnetic resonance imaging (MRI), which is both time-consuming and specialised. To address this, several studies have been looking for biomarkers that could support diagnosis and reflect the disease’s underlying pathology.

Some inflammatory markers, such as C-reactive protein (CRP), interleukin-6 (IL-6), homocysteine, and procalcitonin, have been associated with MRI features, including white matter hyperintensities (WMHs), lacunar infarcts, enlarged perivascular spaces (PVSs), and cerebral microbleeds (CMBs) [5,6,7,8,9,10,11,12,13]. Unfortunately, these tests are not routinely available, are expensive, and may not be accessible in many hospitals in some limited-resource countries. This has led to growing interest in using simpler, routine blood test results, such as neutrophil, lymphocyte, and platelet counts, as alternative indicators. Neutrophils are known to worsen inflammation and tissue damage after stroke, whereas lymphocytes, particularly regulatory T and B cells, appear to play a protective role [14]. Studies have also found that high ratios like platelet-to-neutrophil or platelet-to-lymphocyte are associated with worse outcomes at three months post-stroke [15], and that higher neutrophil counts and platelet-to-neutrophil ratios may increase early mortality [16]. Moreover, an elevated neutrophil-to-lymphocyte ratio (NLR) has been identified as a risk factor for post-stroke pneumonia [17]. Whilst these findings are promising, research specifically looking at the association between these simple inflammatory markers and CSVD remains limited.

Some previous studies have explored whether there is a link between simple inflammatory markers and CSVD, but the results have been contradictory and inconclusive. A study in South Korea found that NLR was associated with large-artery atherosclerosis (LAA), but not with small-vessel occlusion (SVO). However, that study examined only two features of CSVD—silent lacunar infarction (SLI) and WMHs—which may not capture the overall picture of CSVD [18]. On the contrary, a study from China found a significant association between NLR and CSVD. Their findings linked high NLR levels to PVSs and increased white matter signals near the ventricles and in deeper brain regions, though they did not find a link with CMBs [19]. These contrasting results raised further questions about whether these inflammatory markers actually help identify CSVD and whether they can offer any insight into patient outcomes.

This study set out with the hypothesis that simple inflammatory markers from routine blood tests—especially the NLR—were associated with MRI findings of CSVD. It was also hypothesised that higher values of these markers reflected a more severe burden of CSVD on neuroimaging and could be associated with worse clinical outcomes in patients with acute ischaemic stroke. These markers, if proven beneficial, might offer a practical and accessible way to support diagnosis and predict recovery in stroke patients.

## 2. Methods

### 2.1. Study Design and Patient Selection

**Figure 1:**
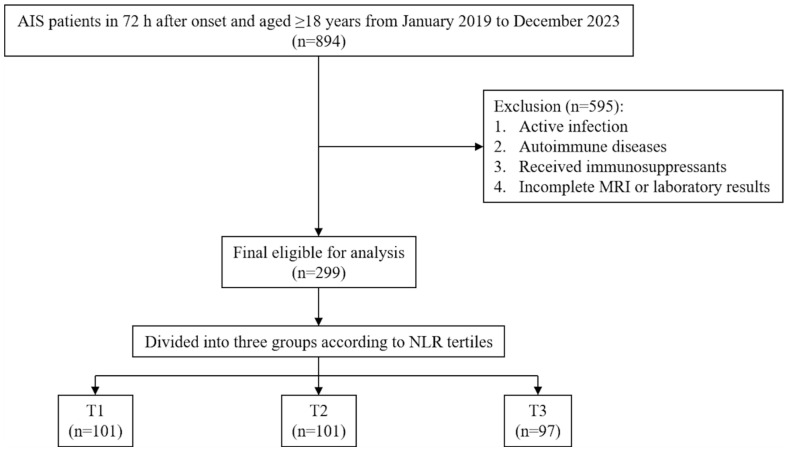
Flowchart of patient selection. AIS, acute ischaemic stroke; MRI, magnetic resonance imaging; NLR, neutrophil–lymphocyte ratio; T, tertile.

This retrospective cohort study was conducted at Maharaj Nakorn Chiang Mai Hospital, a tertiary centre and university-affiliated hospital of Chiang Mai University, Chiang Mai, Thailand. The study enrolled patients aged 18 years or older who were admitted to the acute stroke unit or an internal medicine ward between January 2019 and December 2023, presenting within 72 h of acute ischaemic stroke onset. Diagnostic evaluations were standardised and included laboratory testing, MRI, and magnetic resonance angiography (MRA); all data were retrieved from electronic medical records (EMR). Exclusion criteria included: a diagnosis of an autoimmune disease, receipt of immunosuppressants within three weeks prior to the stroke, or an active infection requiring antibiotics during admission. Patients with incomplete imaging (MRI or MRA) or missing laboratory data for basic inflammatory markers (e.g., complete blood count (CBC), liver function test, and lipid profile) were also excluded. Figure 1 illustrates the participant selection flowchart.

### 2.2. Clinical Data and Functional Outcomes

Demographic and clinical characteristics of the study population were collected from the EMR. Demographic information included age, sex, body mass index (BMI), comorbidities (hypertension, diabetes, dyslipidemia, coronary artery disease, congestive heart failure, atrial fibrillation, and previous stroke), medication used, smoking and alcohol status. Information about ischaemic stroke included systolic blood pressure (SBP), National Institutes of Health Stroke Scale (NIHSS), and modified Rankin Scale (mRS) at admission, classification of acute ischaemic stroke subtype according to the Trial of Org 10,172 in Acute Stroke Treatment (TOAST) [20], endovascular treatment, and medical treatments such as intravenous thrombolysis (IVT) and oral antithrombotics. Functional outcomes were assessed using mRS at discharge and at 90 days, and stroke mortality was also determined at 90 days.

### 2.3. Laboratory and NLR

CBC, liver function test, and lipid profile were collected from all stroke patients on the day of admission and sent to the hospital’s central laboratory for analysis. Because this study included acute ischaemic stroke (AIS) patients who received IVT, blood collection was performed before the treatment to alleviate the major confounder from IVT, as thrombolytic therapy could substantially alter white blood cell count. Estimated neutrophil and lymphocyte counts were derived from the differential count of complete blood cell counts, expressed as a proportion of the total WBC count. The NLR was defined as the ratio of neutrophils to lymphocytes [21].

### 2.4. MRI Acquisition Protocols

All included study populations have a standardised brain MRI and MRA as part of clinical care. The MRI protocol on 3 Tesla SIGNA™ Pioneer (GE HealthCare, Waukesha, Wisconsin, USA) with 21-channel head and neck coils for patients who admitted and followed up at our institute included T1-weighted, T2-weighted (matrix size 512 × 512, slice thickness 4 mm, echo time (TE) 85, repetition time (TR) 6500), axial fluid-attenuated inversion recovery (FLAIR; matrix 384 × 256, slice thickness 4 mm, TE 102, TR 9000, inversion time (TI) 2500), diffusion-weighted imaging (DWI; matrix 100 × 160, slice thickness 4 mm, TE 75, TR 6000, B values 0 and 1000), and susceptibility-weighted imaging (SWI; matrix 384 × 296, slice thickness 2.0 mm, TE 30, TR 38).

### 2.5. Assessment of Neuroimaging Markers of CSVD

The evaluation of neuroimaging markers of CSVD-related lesions was conducted according to the terminology and criteria outlined in the Standards for Reporting Vascular Changes on Neuroimaging 2 (STRIVE-2) [22], along with validated, accepted rating tools and scales for particular CSVD markers. These included haemorrhagic and non-haemorrhagic neuroimaging markers, and the severity of the CSVD burden for each marker was categorised into two or three groups based on previously established total small-vessel disease (SVD) scores [23].

The Microbleed Anatomical Rating Scale (MARS) was utilised as a reliable tool to map brain microbleeds into infratentorial, deep and lobar types [24]. A level of 5 or more lobar CMBs represented a significant CMB load, according to the cerebral amyloid angiopathy (CAA) total SVD score derived from a study utilising a neuropathologic CAA cohort [23]. Cortical superficial siderosis (cSS) was initially classified as none, focal (1–3 sulci), and disseminated (≥4) cSS [25] and further quantified by the cSS multifocality rating scale (score of 0–4) [26].

The Fazekas scale was used to assess deep white matter hyperintensities (DWMHs) and periventricular white matter hyperintensities (PVWMHs) [27]. Confluent DWMHs (Fazekas grade 2 or 3) and irregular PVWMHs extending into deep white matter (Fazekas grade 3) were considered severe WMHs. PVSs in both the basal ganglia (BG-PVSs) and centrum semiovale (CSO-PVSs) were classified using the following scale: 0 for no PVS, 1 for 1–10 PVSs, 2 for 11–20 PVSs, 3 for 21–40 PVSs, and 4 for more than 40 PVSs, where PVS scores of 3–4 corresponded with severe PVSs. Of note, this grading pertains to PVSs on one hemisphere of the brain; a higher score was utilised in cases of asymmetry between the hemispheres [28].

All MRIs evaluating neuroimaging markers of CSVD were rated by a neurologist (N.T.) and a trained vascular neurologist (K.T.), with a random sample of 30 cases (10%) selected for additional review to assess interrater reliability. Discrepancies or inconsistencies among the raters were resolved through consensus discussion with a third party, a senior neuroradiologist (W.W.). Either the kappa statistic or the intraclass correlation coefficient (ICC) was implemented to determine interrater reliability, as appropriate [29,30]. The level of agreement among the raters was substantial, as outlined in detail in Supplementary Table S1.

### 2.6. Statistical Analysis

Descriptive statistics were used to present demographic data, clinical characteristics, and laboratory findings. Categorical variables were presented as numbers and proportions, whilst continuous variables were expressed as either mean ±standard deviation (SD) or median with interquartile range (IQR), depending on the distribution of the data. Differences between groups were analysed using Pearson’s chi-square (χ^2^) test or Fisher’s exact test for categorical variables, based on the sample size within 2 × 2 tables. Comparisons between groups of continuous variables were performed using analysis of variance (ANOVA) or the Kruskal–Wallis test as appropriate. To examine the association between inflammatory markers and neuroimaging markers of CSVD in patients with acute ischaemic stroke, univariable and multinomial logistic regression analyses were performed, whilst adjusting for potential confounding factors, including age, comorbidities, admission NIHSS, and CSVD markers. The results were reported as crude and adjusted odds ratios (ORs) with 95% confidence intervals (CIs) and corresponding *p* values. A *p*-value < 0.05 was considered statistically significant. All statistical analyses were performed using licensed Stata statistical software version 16.1 (Stata Statistical Software: Release 16.1, Stata Corporation, College Station, TX, USA, 2019).

## 3. Results

### 3.1. Characteristics and Demographic Data

A total of 299 AIS patients who met the inclusion criteria were included in the study and divided into three groups according to NLR tertiles: T1 = 0.53–1.74 (*n* = 101), T2 = 1.75–3.04 (*n* = 101), T3 = 3.05–20.17 (*n* = 97). The mean age in this population was 65.7 years (±13.8) with no significant difference among the three groups, and 55.5% were male. The highest prevalence of comorbidities was hypertension (70.6%), followed by dyslipidemia (52.2%), diabetes (27.1%), and previous stroke (26.1%). The prevalence of diabetes was highest in the T3 group (41.2%) compared with the T2 (22.8%) and T1 (17.8%) groups (*p* = 0.001). The median NIHSS score at admission was 3 (2, 4), and the median mRS score was 3 (2, 3), with significant differences between the groups (*p* = 0.005 and <0.001, respectively). According to the TOAST classification, SVO was the most common stroke subtype (59.2%), followed by LAA (24.8%) and cardioembolism (8.7%). Intravenous thrombolysis was administered in 12.0% of patients, whereas mechanical thrombectomy was performed in 0.3% of the selected subjects with no statistical difference. Details of the data collection are presented in Table 1.

### 3.2. CSVD and Functional Outcomes in Different Tertiles of NLR

Focusing on haemorrhagic markers, cSS was rare in our study population, accounting for 1.0%, and mainly was focal cSS (1–3 sulci). cSS multifocality rating scale was graded as 2 in 0.7%. One or more deep CMBs were observed in 17.1% of participants overall, with no significant difference across tertiles. A high lobar CMB burden (≥5 CMBs) was more common in AIS patients in the T3 group, although this difference was not statistically significant (*p* = 0.13). Regarding non-haemorrhagic markers, lacunes and deep lacunes were documented in 47.8% and 32.8% of patients, respectively. Median BG-PVSs was 1 (0, 2) and CSO-PVSs was 1 (1, 3). Severe PVSs, graded 3–4 on a validated four-point scale for enlarged PVSs, were observed in 10.4% of BG-PVSs and 27.4% of CSO-PVSs, with no statistical difference among the three tertiles of NLR (*p* = 0.56 and 0.27, respectively). Severe WMHs were infrequent as DWMHs scored 2–3 in 9.0% and PVWMHs scored 3 in 17.4% (*p* = 0.19 and 0.77, respectively). The median total SVD score was 1 (0, 2), with approximately one-third (32.8%) of participants scoring two or more. All CSVD markers in detail are illustrated in Table 2. Additionally, the cumulative data of each CSVD marker rated in the present study are displayed in Supplementary Table S2.

Concerning the functional outcomes of the subjects in the study, AIS patients in the highest tertile of NLR had a higher median discharge mRS score of 2 (1, 3), compared with 1 (0, 2) and 1 (0, 3) in the T2 and T1 groups, respectively (*p* = 0.01). This corresponded to baseline stroke severity, as evidenced by a higher admission NIHSS score in this group compared to subjects in T2 and T3 (3 (2, 5) vs. 2 (1, 3) and 3 (2, 4); *p* = 0.005, respectively). Long-term functional outcomes, which are more clinically meaningful in stroke outcomes, were comparable across the study population, as measured by 90-day mRS and mortality (Table 2).

### 3.3. Associations of NLR with CSVD and Functional Outcomes

**Figure 2:**
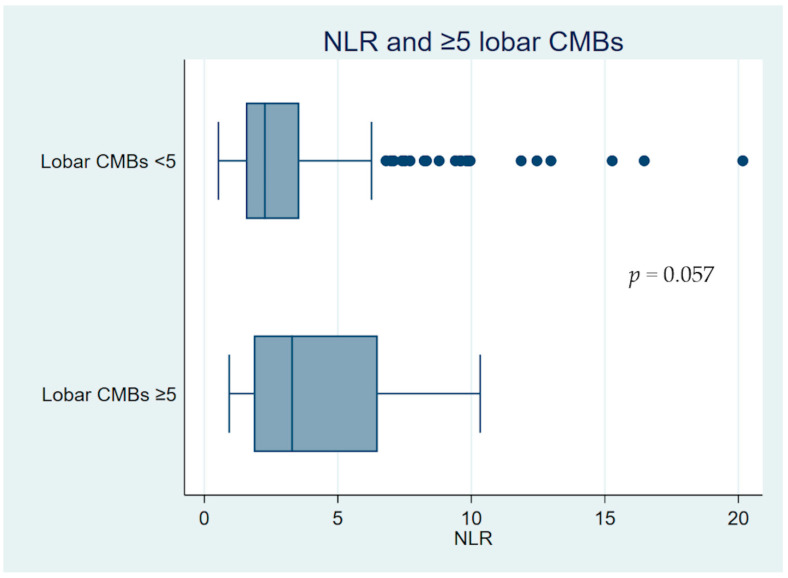
Box plot of admission NLR and lobar CMBs. AIS patients with higher lobar CMB load tended to have higher NLR, with an absence of statistical significance at a threshold level of 0.05. A statistical test produced a *p*-value from the Mann–Whitney U test. AIS, acute ischaemic stroke; CMBs, cerebral microbleeds; NLR, neutrophil–lymphocyte ratio.

In the multinomial regression analysis, there was a higher risk in AIS patients with the T3 group to have five or more lobar CMBs in an unadjusted model (relative risk ratio (RRR) 5.69, 95% CI 1.21–26.68, *p* = 0.03), although there was no statistical significance when adjusted for age, comorbidities, and admission NIHSS score (RRR 3.86; 95% CI 0.79–18.89; *p* = 0.10). Figure 2 illustrates a box plot of admission NLR and lobar CMBs. AIS patients with a higher lobar CMB load tended to have a higher NLR, with marginal non-statistical significance (*p* = 0.057). No significant associations were found between NLR tertiles and other CSVD markers, except for a reduced likelihood of total SVD score ≥ 2 in T2 versus T1 in the fully adjusted model (RRR 0.51, 95% CI 0.26–0.98, *p* = 0.04).

**Figure 3:**
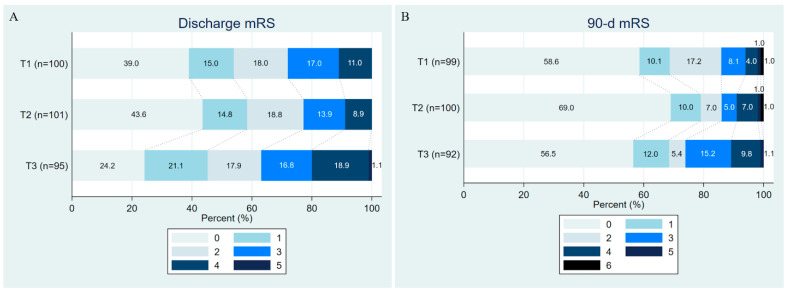
Functional outcomes of the study population. (**A**). Discharge mRS; (**B**) 90-day mRS. Patients in the highest tertile seem to have worse discharge mRS, which is predominantly influenced by initial stroke severity, as indicated by increased NIHSS on admission in the highest NLR tertile. On the contrary, long-term functional outcome at 90 days, which is clinically relevant, does not differ across NLR tertiles. mRS, modified Rankin Scale; NIHSS, National Institutes of Health Stroke Scale; NLR, neutrophil–lymphocyte ratio; T, tertile.

For functional outcomes, being included in the T2 group was associated with greater odds of achieving a 90-day mRS 0–1 compared with the T1 group (RRR 2.16, 95% CI 1.05–4.44, *p* = 0.04) after full adjustment for potential confounders. No significant associations were observed between NLR tertiles and favourable functional outcomes at discharge or 90-day mortality. Details of the associations between NLR and CSVD, as well as with functional outcomes, are presented in Table 3. Figure 3 shows a stacked bar chart of the distribution of functional outcomes for the study population at discharge (Figure 3A) and at 90 days (Figure 3B).

## 4. Discussion

In this single-centre retrospective cohort study, we examined the associations between blood NLR tertiles, a simple biomarker of circulating subclinical inflammation, and a broad range of CSVD neuroimaging markers, as well as short- and long-term functional outcomes in AIS patients. In the present study, we found that higher NLR on admission, especially in the highest tertile (>3.05), was associated with a greater burden of lobar CMBs in univariable multinomial logistic regression, a particular haemorrhagic marker of CSVD. This supports the idea that systemic inflammation plays a significant role in CSVD and aligns with previous studies demonstrating the potential of blood NLR as a predictor of CSVD [21,31]. Although associations between this peripheral inflammatory marker and functional outcomes following AIS were not established in our study, our findings extend to the SVD spectrum, as previous research has found that inflammatory markers are associated with stroke severity and poor outcomes [32,33].

Inflammation is known to damage the endothelium, reduce vascular reactivity, and disrupt the blood–brain barrier (BBB), all of which are thought to contribute to CSVD [1]. Since CSVD doubles the risk of stroke and is a significant cause of long-term disability, dementia, and cognitive decline [3,4], having a simple blood biomarker like NLR could be helpful in risk assessment in routine clinical practice.

Previous studies have reported associations between inflammatory markers such as CRP, IL-6, and procalcitonin and MRI features of CSVD, including WMHs, lacunes, and PVSs [5,6,7,8,9,10,11,12,13]. However, these biomarkers are not routinely measured in many centres and may be less accessible in resource-limited settings. In contrast, NLR can be derived from a standard complete blood count and is widely available in patients with AIS. Neutrophils and lymphocytes are thought to play differing roles in post-stroke inflammatory responses, and NLR has been proposed as a general indicator of systemic inflammatory balance [14]. Although this framework may offer a biological context for our findings, the observed relationship between NLR and lobar CMB burden in our cohort should be interpreted cautiously, as it was not robust after adjustment for confounding factors. Furthermore, the lack of consistent associations between NLR and other key CSVD markers suggests that NLR may not reliably reflect the overall CSVD burden in this population. These findings indicate that whilst systemic inflammation is a known factor in cerebrovascular pathology, the role of NLR as a comprehensive imaging biomarker for CSVD remains uncertain and requires further validation through larger longitudinal studies.

Based on the disease mechanisms of CSVD, several hypotheses are proposed and tested to elucidate the underlying pathophysiology of the immune system and CSVD. Vessel wall damage and microleakage are the primary mechanisms underlying CMB formation [34]. Chronic subclinical inflammation, as measured by blood NLR, promotes WBC adherence to the vascular endothelium, leading to endothelial dysfunction, triggering inflammatory cascades, and ultimately leading to the development of CSVD lesions [35,36]. Our results on a potential increase in lobar CMB load and an increased NLR level extended scientific knowledge on neuroinflammation and CSVD, as previous studies have established associations or correlations with most nonhaemorrhagic markers, specifically WMHs, PVSs, lacunes, and recent small cortical infarct (RSSI) [21,31,35,37]. A recent systematic review of 18 studies highlighted the emerging role of circulating immune cells in the pathology of CSVD and reported a positive association between NLR and the monocyte-to-high-density-lipoprotein (HDL) ratio (MHR) with various CSVD markers [36]. Additionally, CMBs, when documented exclusively in a lobar distribution, are a neuroimaging hallmark of CAA; findings from our cohort could generate research hypotheses on the associations between altered peripheral inflammation and the pathogenesis of CAA. Our study adds to this debate by showing an association between lobar CMBs and the broader STRIVE-2 neuroimaging criteria [22]. Taken together, these findings suggest that inflammation may affect CSVD features differently. However, it is important to acknowledge that NLR was measured only during the acute phase of stroke, and alterations in the WBC profile might have developed concurrently with acute ischemic injury. Consequently, it is uncertain whether an increased NLR indicates pre-existing CSVD, acute stroke severity, or systemic stress.

The observed trend toward worse discharge functional status in patients with higher NLR appeared to be largely influenced by baseline stroke severity, as individuals in the highest NLR tertile had higher admission NIHSS scores, and the association was attenuated after adjustment. However, this was consistent with earlier reports showing that high inflammatory ratios predict poor recovery and higher mortality after stroke [15,16,17]. A study investigating the association between NLR and functional outcome in patients with a single small subcortical infarct (SSSI) demonstrated that higher NLR levels were associated with unfavourable functional outcomes at discharge and at 90 days, after adjusting for confounding variables [38]. Significantly, the NLR value was independently associated with early neurological deterioration (END) in patients with single subcortical infarction and diabetes, and was identified as an independent predictor of END [39]. These findings are consistent with those of the present study, which showed that increased NLR was observed in AIS patients with diabetes and that these patients had a higher proportion of high discharge mRS scores. It can be explained by the underlying mechanism that low-level chronic inflammation is accompanied by multiple vascular risk factors for CSVD, such as hypertension and diabetes. Interestingly, patients in the middle NLR tertile in our study seemed to recover better than those in the lowest tertile. This might be explained by the paradoxical function of regulatory T cells (Tregs) in ischaemic stroke. Normally, Tregs could prevent ischaemic damage to the brain by regulating the inflammatory process. On the other hand, by inducing microvascular abnormalities, they exacerbated the insult [40]. The isolated finding that the middle NLR tertile was associated with a more favourable 90-day outcome lacks a definitive biological explanation—though potentially related to the complex, non-linear role of regulatory T cells—and should be interpreted cautiously as a possible chance finding. Additionally, more clinically relevant long-term outcomes, including 90-day mRS and mortality, did not show a consistent or graded relationship with NLR, suggesting that this marker does not have a robust prognostic role in our cohort. All of these suggest that the neuroinflammatory response in CSVD is complicated and warrants further investigation.

The present study comprehensively examined the full range of neuroimaging markers of CSVD, clearly delineated haemorrhagic and non-haemorrhagic markers, and their association with circulating inflammatory markers in AIS populations, using the latest neuroimaging standards for SVD research, STRIVE-2, and incorporating several validated rating scales for CSVD, namely MARS, cSS and Fazekas visual rating scale, and PVS semiquantitative scale. However, we acknowledge some limitations to the present study. Our study was retrospective and conducted at a single centre among a single ethnic group. As ischaemic stroke might differ across diverse populations, particularly in SVO, generalisability may be limited. Second, NLR was measured only at admission and could not be followed to assess changes over time. Therefore, this might be insufficient to capture the alterations along the course of ischaemic insults. CSVD is a longstanding and cumulative condition, whereas NLR serves as a dynamic, short-term indicator of inflammation. The absence of longitudinal NLR information constrains mechanistic understanding. Finally, the number of patients with severe WMHs or cSS was small in our study; consequently, this might limit the power to examine associations with these features. Further prospective research at follow-up time points, specifically examining various circulating immune cell markers, including MHR and the lymphocyte-to-monocyte ratio (LMR), might add value to these systemic immune status markers in CSVD and their outcomes or prognosis.

## 5. Conclusions

In this retrospective cohort of patients with acute ischaemic stroke, admission NLR showed a possible association with a higher burden of lobar cerebral microbleeds in unadjusted analyses, although this relationship was attenuated after accounting for confounders. No consistent associations were observed between NLR and most other neuroimaging markers of cerebral small vessel disease. The relationship between NLR and functional outcomes was also not uniform across tertiles and should be interpreted cautiously.

Taken together, our findings suggest that NLR may reflect aspects of systemic inflammation relevant to cerebrovascular pathology; however, its role as an imaging or prognostic marker in cerebral small vessel disease remains uncertain. These results should be considered exploratory and hypothesis-generating. Larger prospective studies with longitudinal biomarker assessments are needed to clarify the relationship between inflammatory indices such as NLR, CSVD burden, and stroke outcomes before any clinical implications can be drawn.

## Figures and Tables

**Table 1:** Baseline characteristics of participants according to the tertiles of neutrophil–lymphocyte ratio.

Characteristics	Neutrophil–Lymphocyte Ratio				*p* Value
	Total (*n* = 299)	T1 (0.53–1.74) (*n* = 101)	T2 (1.75–3.04) (*n* = 101)	T3 (3.05–20.17) (*n* = 97)	
Patient characteristics					
Age, years—mean (SD)	65.7 (13.8)	64.6 (13.2)	67.9 (14.5)	64.5 (13.4)	0.15
Male—*n* (%)	166 (55.5)	55 (54.5)	55 (54.5)	56 (57.7)	0.87
BMI, kg/m^2^—mean (SD)	24.3 (3.9)	24.3 (3.4)	24.0 (4.3)	24.5 (4.2)	0.71
Smoking—*n* (%)	30 (10.0)	12 (11.9)	9 (8.9)	9 (9.3)	0.75
Alcohol drinking—*n* (%)	68 (22.7)	27 (26.7)	20 (19.8)	21 (21.7)	0.48
Comorbidities—*n* (%)					
Hypertension	211 (70.6)	65 (64.4)	74 (73.3)	72 (74.2)	0.24
Diabetes	81 (27.1)	18 (17.8)	23 (22.8)	40 (41.2)	0.001
Dyslipidemia	156 (52.2)	50 (49.5)	55 (54.5)	51 (52.6)	0.78
Coronary artery disease	17 (5.7)	6 (5.9)	4 (4.0)	7 (7.2)	0.61
Congestive heart failure	1 (0.3)	0 (0)	0 (0)	1 (1.0)	0.32
Atrial fibrillation	18 (6.0)	7 (6.9)	7 (6.9)	4 (4.1)	0.65
Previous stroke	78 (26.1)	22 (21.8)	26 (25.7)	30 (30.9)	0.34
Medication—*n* (%)					
Antihypertensive drugs	172 (57.5)	54 (53.5)	59 (58.4)	59 (60.8)	0.56
Oral hypoglycaemic agents	62 (20.7)	12 (11.9)	21 (20.8)	29 (29.9)	0.008
Statin	156 (52.2)	45 (44.6)	59 (58.4)	52 (53.6)	0.14
Antiplatelets	101 (33.8)	28 (27.7)	38 (37.6)	35 (36.1)	0.28
Oral anticoagulants	9 (3.0)	2 (2.0)	4 (4.0)	3 (3.1)	0.78
Stroke information					
Admission SBP, mmHg—mean (SD)	155.5 (30.1)	157.0 (27.0)	156.9 (32.3)	152.4 (30.8)	0.46
Admission NIHSS—median (IQR)	3 (2, 4)	2 (1, 3)	3 (2, 4)	3 (2, 5)	0.005
Admission mRS—median (IQR)	3 (2, 3)	3 (2, 3)	3 (1, 3)	3 (2, 4)	<0.001
TOAST classification—*n* (%)					0.28
Large-artery atherosclerosis	74 (24.8)	29 (28.7)	18 (17.8)	27 (27.8)	
Cardioembolism	26 (8.7)	8 (7.9)	12 (11.9)	6 (6.2)	
Small-vessel occlusion (lacune)	177 (59.2)	59 (58.4)	59 (58.4)	59 (60.8)	
Other determined	9 (3.0)	3 (3.0)	4 (4.0)	2 (2.1)	
Undetermined	13 (4.3)	2 (2.0)	8 (7.9)	3 (3.1)	
Reperfusion therapy—*n* (%)					
Intravenous thrombolysis	36 (12.0)	14 (13.9)	11 (10.9)	11 (11.3)	0.78
Mechanical thrombectomy	1 (0.3)	0 (0)	1 (1.0)	0 (0)	1.00
Laboratory—mean (SD)					
Platelet count, cells/mm^3^	243,364 (74,986)	238,584 (65,198)	255,366 (82,018)	235,845 (76,069)	0.14
Absolute neutrophil count, cells/mm^3^	5045 (2265)	3529 (940)	4804 (1485)	6873 (2618)	<0.001
Absolute lymphocyte count, cells/mm^3^	2071 (968)	2784 (1026)	2079 (633)	1319 (540)	<0.001
Albumin, mg/dL	4.1 (0.4)	4.2 (0.3)	4.1 (0.4)	4.0 (0.5)	0.047
HDL-C, mg/dL	48.3 (13.7)	47.7 (11.8)	48.7 (14.6)	48.6 (14.5)	0.84

Abbreviations: BMI, body mass index; HDL-C, high-density lipoprotein cholesterol; IQR, interquartile range; mRS, modified Rankin Scale; NIHSS, National Institute of Health Stroke Scale; SBP, systolic blood pressure; SD, standard deviation; TOAST, Trial of Org 10,172 in Acute Stroke Treatment.

**Table 2:** Cerebral small vessel disease markers and functional outcomes in different tertiles of neutrophil–lymphocyte ratio.

	Neutrophil–Lymphocyte Ratio				*p* Value
	Total (*n* = 299)	T1 (0.53–1.74) (*n* = 101)	T2 (1.75–3.04) (*n* = 101)	T3 (3.05–20.17) (*n* = 97)	
CSVD markers					
cSS ^†^—no. (%)	3 (1.0)	1 (1.0)	1 (1.0)	1 (1.0)	1.00
None	296 (99.0)	100 (99.0)	100 (99.0)	96 (99.0)	
Focal (1–3 sulci)	3 (1.0)	1 (1.0)	1 (1.0)	1 (1.0)	
Deep CMBs—median (IQR)	0 (0, 0)	0 (0, 0)	0 (0, 0)	0 (0, 0)	0.93
≥1 CMBs—no. (%)	51 (17.1)	18 (17.8)	16 (15.8)	17 (17.5)	0.92
Lobar CMBs—median (IQR)	0 (0, 0)	0 (0, 0)	0 (0, 0)	0 (0, 0)	0.37
0–1 CMB—no. (%)	267 (89.3)	94 (93.0)	89 (88.2)	84 (86.6)	0.13
2–4 CMBs—no. (%)	14 (4.7)	5 (5.0)	6 (5.9)	3 (3.1)	
≥5 CMBs—no. (%)	18 (6.0)	2 (2.0)	6 (5.9)	10 (10.3)	
Lacunes					
Lacunes (≥1 lacunes)—no. (%)	143 (47.8)	44 (43.6)	45 (44.6)	54 (55.7)	0.17
Deep lacunes (≥1 lacunes)—no. (%)	98 (32.8)	27 (26.7)	36 (35.6)	35 (36.1)	0.28
BG-PVSs, 0–4—median (IQR)	1 (0, 2)	1 (0, 2)	1 (0, 2)	1 (0, 2)	0.91
0–2—no. (%)	268 (89.6)	91 (90.1)	88 (87.1)	89 (91.8)	0.56
3–4—no. (%)	31 (10.4)	10 (9.9)	13 (12.9)	8 (8.2)	
CSO-PVSs, 0–4—median (IQR)	1 (1, 3)	2 (1, 3)	1 (1, 3)	1 (0, 2)	0.08
0–2—no. (%)	217 (72.6)	72 (71.3)	69 (68.3)	76 (78.4)	0.27
3–4—no. (%)	82 (27.4)	29 (28.7)	32 (31.7)	21 (21.6)	
DWMHs, 0–3—median (IQR)	0 (0, 1)	0 (0, 0)	0 (0, 1)	0 (0, 1)	0.16
0–1—no. (%)	272 (91.0)	94 (93.1)	94 (93.1)	84 (86.6)	0.19
2–3—no. (%)	27 (9.0)	7 (6.9)	7 (6.9)	13 (13.4)	
PVWMHs, 0–3—median (IQR)	1 (1, 2)	1 (0, 2)	1 (1, 2)	1 (1, 2)	0.10
0–2—no. (%)	247 (82.6)	84 (83.2)	85 (84.2)	78 (80.4)	0.77
3—no. (%)	52 (17.4)	17 (16.8)	16 (15.8)	19 (19.6)	
Total SVD score,—median (IQR)	1 (0, 2)	1 (0, 2)	1 (0, 2)	1 (0, 2)	0.44
≥2—no. (%)	98 (32.8)	35 (34.7)	28 (27.7)	35 (36.1)	0.40
Functional outcomes					
Discharge mRS—median (IQR)	1 (0, 3)	1 (0, 3)	1 (0, 2)	2 (1, 3)	0.01
90-day mRS—median (IQR)	0 (0, 2)	0 (0, 2)	0 (0, 1)	0 (0, 3)	0.16
90-day mortality—no. (%)	4 (1.4)	1 (1.0)	1 (1.0)	2 (2.1)	0.70

Abbreviations: BG-PVS, basal ganglia perivascular space; CMB, cerebral microbleed; CSO-PVS, centrum semiovale perivascular space; cSS, cortical superficial siderosis; CSVD, cerebral small vessel disease; DWMH, deep white matter hyperintensity; IQR, interquartile range; mRS, modified Rankin Scale; PVWMH, periventricular white matter hyperintensity; SD, standard deviation; SVD, small vessel disease. ^†^ No disseminated cSS was observed in the study participants.

**Table 3:** Multinomial logistic regression analysis between tertiles of admission neutrophil–lymphocyte ratio and cerebral small vessel disease markers and functional outcomes.

Outcomes	Unadjusted RRR (95% CI)	*p* Value	Model 1 Adjusted RRR (95% CI) ^†^	*p* Value	Model 2 Adjusted RRR (95% CI) ^‡^	*p* Value
CSVD markers						
Deep CMB ≥ 1						
T1	Reference	NA	Reference	NA	Reference	NA
T2	0.87 (0.41–1.82)	0.71	0.82 (0.39–1.72)	0.60	0.71 (0.33–1.55)	0.39
T3	0.98 (0.47–2.03)	0.96	0.78 (0.36–1.69)	0.53	0.58 (0.24–1.36)	0.21
Lobar CMBs ≥ 5						
T1	Reference	NA	Reference	NA	Reference	NA
T2	3.13 (0.62–15.87)	0.17	2.77 (0.54–14.34)	0.22	NA	NA
T3	5.69 (1.21–26.68)	0.03	3.86 (0.79–18.89)	0.10	NA	NA
Lacune ≥ 1						
T1	Reference	NA	Reference	NA	Reference	NA
T2	1.04 (0.60–1.81)	0.89	0.92 (0.52–1.63)	0.77	0.87 (0.48–1.55)	0.63
T3	1.63 (0.93–2.85)	0.09	1.52 (0.83–2.77)	0.18	1.41 (0.77–2.59)	0.27
Deep lacune ≥ 1						
T1	Reference	NA	Reference	NA	Reference	NA
T2	1.52 (0.83–2.77)	0.17	1.45 (0.78–2.67)	0.24	1.40 (0.76–2.59)	0.29
T3	1.55 (0.84–2.83)	0.16	1.46 (0.77–2.76)	0.24	1.39 (0.73–2.64)	0.32
BG-PVSs score 3–4						
T1	Reference	NA	Reference	NA	Reference	NA
T2	1.34 (0.56–3.22)	0.51	1.13 (0.46–2.79)	0.79	1.09 (0.44–2.72)	0.85
T3	0.82 (0.31–2.17)	0.69	0.94 (0.34–2.62)	0.90	0.85 (0.30–2.45)	0.77
CSO-PVSs score 3–4						
T1	Reference	NA	Reference	NA	Reference	NA
T2	1.15 (0.63–2.10)	0.65	1.02 (0.55–1.89)	0.96	1.03 (0.56–1.92)	0.92
T3	0.69 (0.36–1.31)	0.25	0.67 (0.34–1.32)	0.24	0.68 (0.34–1.35)	0.27
DWMHs score 2–3						
T1	Reference	NA	Reference	NA	Reference	NA
T2	1.00 (0.34–2.96)	1.00	0.78 (0.25–2.45)	0.67	0.70 (0.22–2.22)	0.54
T3	2.08 (0.79–5.45)	0.14	1.77 (0.63–4.98)	0.28	1.52 (0.53–4.39)	0.43
PVWMHs score 3						
T1	Reference	NA	Reference	NA	Reference	NA
T2	0.93 (0.44–1.96)	0.85	0.66 (0.30–1.49)	0.32	0.58 (0.25–1.34)	0.20
T3	1.20 (0.58–2.48)	0.62	1.03 (0.46–2.33)	0.93	0.87 (0.37–2.01)	0.74
Total SVD score ≥ 2						
T1	Reference	NA	Reference	NA	Reference	NA
T2	0.72 (0.40–1.32)	0.29	0.59 (0.31–1.11)	0.10	NA	NA
T3	1.06 (0.59–1.91)	0.83	0.92 (0.48–1.74)	0.79	NA	NA
Functional outcomes						
Discharge mRS 0–1						
T1	Reference	NA	Reference	NA	Reference	NA
T2	1.20 (0.68–2.09)	0.53	1.28 (0.69–2.37)	0.43	1.31 (0.71–2.43)	0.39
T3	0.70 (0.40–1.24)	0.22	0.92 (0.49–1.74)	0.81	0.97 (0.51–1.82)	0.91
90-day mRS 0–1						
T1	Reference	NA	Reference	NA	Reference	NA
T2	1.71 (0.90–3.26)	0.10	2.08 (1.02–4.25)	0.045	2.16 (1.05–4.44)	0.04
T3	0.99 (0.54–1.83)	0.98	1.63 (0.80–3.33)	0.18	1.73 (0.84–3.56)	0.14
90-day mortality						
T1	Reference	NA	Reference	NA	Reference	NA
T2	0.99 (0.06–16.05)	0.99	0.65 (0.04–10.94)	0.77	0.60 (0.03–10.26)	0.72
T3	2.11 (0.19–23.62)	0.55	1.29 (0.10–16.46)	0.84	1.11 (0.08–15.14)	0.94

Abbreviations: BG-PVS, basal ganglia perivascular space; CI, confidence interval; CMB, cerebral microbleed; CSO-PVS, centrum semiovale perivascular space; CSVD, cerebral small vessel disease; DWMH, deep white matter hyperintensity; mRS, modified Rankin Scale; NA, not applicable; NLR, neutrophil–lymphocyte ratio; PVWMH, periventricular white matter hyperintensity; RRR, relative-risk ratio; SVD, small vessel disease; T, tertile. ^†^ Adjusted for age, diabetes, and admission NIHSS. ^‡^ Adjusted for age, diabetes, and admission NIHSS and CSVD marker, including lobar CMBs ≥ 5. The adjustments for other CVSD markers have been applied where appropriate.

## Data Availability

The datasets generated and/or analysed during the current study are available from the corresponding author on reasonable request.

## Supplementary Materials

The following supporting information can be downloaded at: https://www.mdpi.com/article/10.3390/life16020337/s1. Table S1: Interrater reliability; Table S2: Cumulative data for each cerebral small vessel disease marker.

## References

[1] Pantoni L. (2010). Cerebral small vessel disease: From pathogenesis and clinical characteristics to therapeutic challenges. Lancet Neurol..

[2] Bernick C. (2001). Silent MRI infarcts and the risk of future stroke: The cardiovascular health study. Neurology.

[3] Pantoni L., Gorelick P.B. (2014). Cerebral Small Vessel Disease.

[4] Zhang X., Tang Y., Xie Y., Ding C., Xiao J., Jiang X., Shan H., Lin Y., Li C., Hu D. (2017). Total magnetic resonance imaging burden of cerebral small-vessel disease is associated with post-stroke depression in patients with acute lacunar stroke. Eur. J. Neurol..

[5] Wersching H., Duning T., Lohmann H., Mohammadi S., Stehling C., Fobker M., Conty M., Minnerup J., Ringelstein E., Berger K. (2010). Serum C-reactive protein is linked to cerebral microstructural integrity and cognitive function. Neurology.

[6] Satizabal C., Zhu Y., Mazoyer B., Dufouil C., Tzourio C. (2012). Circulating IL-6 and CRP are associated with MRI findings in the elderly: The 3C-Dijon Study. Neurology.

[7] Aribisala B.S., Wiseman S., Morris Z., Valdés-Hernández M.C., Royle N.A., Maniega S.M., Gow A.J., Corley J., Bastin M.E., Starr J. (2014). Circulating inflammatory markers are associated with magnetic resonance imaging-visible perivascular spaces but not directly with white matter hyperintensities. Stroke.

[8] Walker K.A., Power M.C., Hoogeveen R.C., Folsom A.R., Ballantyne C.M., Knopman D.S., Windham B.G., Selvin E., Jack C.R., Gottesman R.F. (2017). Midlife systemic inflammation, late-life white matter integrity, and cerebral small vessel disease: The atherosclerosis risk in communities study. Stroke.

[9] Gu Y., Gutierrez J., Meier I.B., Guzman V.A., Manly J.J., Schupf N., Brickman A.M., Mayeux R. (2018). Circulating inflammatory biomarkers are related to cerebrovascular disease in older adults. Neurol. Neuroimmunol. Neuroinflamm..

[10] Hilal S., Ikram M.A., Verbeek M.M., Franco O.H., Stoops E., Vanderstichele H., Niessen W.J., Vernooij M.W. (2018). C-reactive protein, plasma amyloid-β levels, and their interaction with magnetic resonance imaging markers. Stroke.

[11] Li G., Zhu C., Li J., Wang X., Zhang Q., Zheng H., Zhan C. (2018). Increased level of procalcitonin is associated with total MRI burden of cerebral small vessel disease in patients with ischemic stroke. Neurosci. Lett..

[12] Arba F., Giannini A., Piccardi B., Biagini S., Palumbo V., Giusti B., Nencini P., Maria Gori A., Nesi M., Pracucci G. (2019). Small vessel disease and biomarkers of endothelial dysfunction after ischaemic stroke. Eur. Stroke J..

[13] Zhang L., Gao F., Zhang Y., Hu P., Yao Y., Zhang Q., He Y., Shang Q., Zhang Y. (2022). Analysis of risk factors for the development of cognitive dysfunction in patients with cerebral small vessel disease and the construction of a predictive model. Front. Neurol..

[14] Low A., Su L., Stefaniak J.D., Mak E., Dounavi M.-E., Muniz-Terrera G., Ritchie K., Ritchie C.W., Markus H.S., O’brien J.T. (2021). Inherited risk of dementia and the progression of cerebral small vessel disease and inflammatory markers in cognitively healthy midlife adults: The PREVENT-Dementia study. Neurobiol. Aging.

[15] Simats A., García-Berrocoso T., Montaner J. (2016). Neuroinflammatory biomarkers: From stroke diagnosis and prognosis to therapy. Biochim. Biophys. Acta (BBA)-Mol. Basis Dis..

[16] Jin P., Li X., Chen J., Zhang Z., Hu W., Chen L., Feng X., Shao B. (2019). Platelet-to-neutrophil ratio is a prognostic marker for 90-days outcome in acute ischemic stroke. J. Clin. Neurosci..

[17] Hu Z.-B., Zhong Q.-Q., Lu Z.-X., Zhu F. (2022). Association of platelet-to-white blood cell ratio and platelet-to-neutrophil ratio with the risk of fatal stroke occurrence in middle-aged to older Chinese. BMC Geriatr..

[18] Wang Q., Liu Y., Han L., He F., Cai N., Zhang Q., Wang J. (2021). Risk factors for acute stroke-associated pneumonia and prediction of neutrophil-to-lymphocyte ratios. Am. J. Emerg. Med..

[19] Chung D., Lee K.O., Choi J.W., Kim N.K., Kim O.J., Kim S.H., Oh S.H., Kim W.C. (2020). Blood Neutrophil/Lymphocyte Ratio Is Associated With Cerebral Large-Artery Atherosclerosis but Not With Cerebral Small-Vessel Disease. Front. Neurol..

[20] Adams H.P., Bendixen B.H., Kappelle L.J., Biller J., Love B.B., Gordon D.L., Marsh E.E. (1993). Classification of subtype of acute ischemic stroke. Definitions for use in a multicenter clinical trial. TOAST. Trial of Org 10172 in Acute Stroke Treatment. Stroke.

[21] Wang Y., Ma L., Zhang M., Wei J., Li X., Pan X., Ma A. (2022). Blood Neutrophil-to-Lymphocyte Ratio as a Predictor of Cerebral Small-Vessel Disease. Med. Sci. Monit..

[22] Duering M., Biessels G.J., Brodtmann A., Chen C., Cordonnier C., de Leeuw F.-E., Debette S., Frayne R., Jouvent E., Rost N.S. (2023). Neuroimaging standards for research into small vessel disease—Advances since 2013. Lancet Neurol..

[23] Charidimou A., Martinez-Ramirez S., Reijmer Y.D., Oliveira-Filho J., Lauer A., Roongpiboonsopit D., Frosch M., Vashkevich A., Ayres A., Rosand J. (2016). Total Magnetic Resonance Imaging Burden of Small Vessel Disease in Cerebral Amyloid Angiopathy: An Imaging-Pathologic Study of Concept Validation. JAMA Neurol.

[24] Gregoire S., Chaudhary U., Brown M., Yousry T., Kallis C., Jager H., Werring D. (2009). The microbleed anatomical rating scale (MARS) reliability of a tool to map brain microbleeds. Neurology.

[25] Linn J., Halpin A., Demaerel P., Ruhland J., Giese A.D., Dichgans M., van Buchem M.A., Bruckmann H., Greenberg S.M. (2010). Prevalence of superficial siderosis in patients with cerebral amyloid angiopathy. Neurology.

[26] Charidimou A., Boulouis G., Roongpiboonsopit D., Auriel E., Pasi M., Haley K., van Etten E.S., Martinez-Ramirez S., Ayres A., Vashkevich A. (2017). Cortical superficial siderosis multifocality in cerebral amyloid angiopathy: A prospective study. Neurology.

[27] Fazekas F., Chawluk J.B., Alavi A., Hurtig H.I., Zimmerman R.A. (1987). MR signal abnormalities at 1.5 T in Alzheimer’s dementia and normal aging. Am. J. Roentgenol..

[28] Doubal F.N., MacLullich A.M., Ferguson K.J., Dennis M.S., Wardlaw J.M. (2010). Enlarged perivascular spaces on MRI are a feature of cerebral small vessel disease. Stroke.

[29] McHugh M.L. (2012). Interrater reliability: The kappa statistic. Biochem. Med..

[30] Koo T.K., Li M.Y. (2016). A Guideline of Selecting and Reporting Intraclass Correlation Coefficients for Reliability Research. J. Chiropr. Med..

[31] Cai J., Zeng X., Huang X., Dong H., Liu J., Lin J., Xie M., Wei X. (2024). Relationship of neutrophil/lymphocyte ratio with cerebral small vessel disease and its common imaging markers. Immun. Inflamm. Dis..

[32] Celikbilek A., Ismailogullari S., Zararsiz G. (2014). Neutrophil to lymphocyte ratio predicts poor prognosis in ischemic cerebrovascular disease. J. Clin. Lab. Anal..

[33] Ferro D., Matias M., Neto J., Dias R., Moreira G., Petersen N., Azevedo E., Castro P. (2021). Neutrophil-to-Lymphocyte Ratio Predicts Cerebral Edema and Clinical Worsening Early After Reperfusion Therapy in Stroke. Stroke.

[34] Wardlaw J.M., Smith C., Dichgans M. (2019). Small vessel disease: Mechanisms and clinical implications. Lancet Neurol..

[35] Jiang L., Cai X., Yao D., Jing J., Mei L., Yang Y., Li S., Jin A., Meng X., Li H. (2022). Association of inflammatory markers with cerebral small vessel disease in community-based population. J. Neuroinflamm..

[36] Van der Taelen L., Briones A.M., Unger T., Staals J., van Oostenbrugge R.J., Foulquier S. (2025). Circulating immune cells in cerebral small vessel disease: A systematic review. Biogerontology.

[37] Hou L., Zhang S., Qi D., Jia T., Wang H., Zhang W., Wei S., Xue C., Wang P. (2022). Correlation between neutrophil/lymphocyte ratio and cognitive impairment in cerebral small vessel disease patients: A retrospective study. Front. Neurol..

[38] Wu Y., Hu J., Zhao Y., Ju D., Cao S., Guo J., Song W., Mo R., Lei S., Wu Y. (2025). The neutrophil-to-lymphocyte ratio is associated with functional outcome among single small subcortical infarction: Mediating effects of white matter hyperintensities. J. Stroke Cerebrovasc. Dis..

[39] Fang L., Wang Y., Zhang H., Jiang L., Jin X., Gu Y., Wu M., Pei S., Cao Y. (2022). The neutrophil-to-lymphocyte ratio is an important indicator correlated to early neurological deterioration in single subcortical infarct patients with diabetes. Front. Neurol..

[40] Xu X., Li M., Jiang Y. (2013). The paradox role of regulatory T cells in ischemic stroke. Sci. World J..

