# Supplementary material for: Associations of Neutrophil-to-Lymphocyte Ratio with Cerebral Small Vessel Disease and Functional Outcome in Acute Ischaemic Stroke Patients
Source: Life (Basel). 2026 Feb 15;16(2):337. doi: 10.3390/life16020337 (PMC12941587; doi:10.3390/life16020337)
Supplement: Supplementary file 1 [file life-16-00337-s001.zip › life-4129999-supplementary.pdf]

Associations of Neutrophil-to-Lymphocyte Ratio with Cerebral Small Vessel Disease and Functional Outcome in Acute Ischaemic Stroke Patients

Supplementary Materials:

- **Table S1.** Interrater reliability.
- **Table S2.** Cumulative data for each cerebral small vessel disease marker.

Table S1. Interrater reliability.

CSVD markers	Agreement (%)	Expected agreement (%)	Kappa	SE	Z	Prob>Z
cSS	96.67	90.44	0.6512	0.1711	3.81	0.0001
cSS multifocality rating scale	93.33	87.22	0.4783	0.1182	4.05	<0.0001
Infratentorial CMB	80.00	70.78	0.3156	0.1372	2.30	0.0107
Deep CMB	86.67	72.00	0.5238	0.1773	2.95	0.0016
Lobar CMB	90.00	78.33	0.5385	0.1251	4.30	<0.0001
Total CMB	86.67	58.78	0.6765	0.1276	5.30	<0.0001
Deep lacune	90.00	52.67	0.7887	0.1821	4.33	<0.0001
Lacune	96.67	50.00	0.9333	0.1822	5.12	<0.0001
BG-PVS	73.33	32.67	0.6040	0.1016	5.95	<0.0001
CSO-PVS	76.67	26.89	0.6809	0.1033	6.59	<0.0001
DWMH	80.00	34.67	0.6939	0.1129	6.14	<0.0001
PVWMH	86.67	76.67	0.4286	0.1750	2.45	0.0072

Abbreviations: BG-PVS, basal ganglia perivascular space; CMB, cerebral microbleed; CSO-PVS, centrum semiovale perivascular space; cSS, cortical superficial siderosis; CSVD, cerebral small vessel disease; DWMH, deep white matter hyperintensity; PVWMH, periventricular white matter hyperintensity; SE, standard error.

Table S2. Cumulative data of each cerebral small vessel disease marker.

cSS multifocality rating scale		Deep CMB		Lobar CMB		Total CMB		BG-PVS		CSO-PVS		DWMH		PVWMH		Total SVD score	
**Scale**	**Freq. (%)**	**No.**	**Freq. (%)**	**No.**	**Freq. (%)**	**No.**	**Freq. (%)**	**Scale**	**Freq. (%)**	**Scale**	**Freq. (%)**	**Scale**	**Freq. (%)**	**Scale**	**Freq. (%)**	**Score**	**Freq. (%)**
0	296 (99.0)	0	248 (82.9)	0	243 (81.3)	0	210 (70.3)	0	96 (32.1)	0	66 (22.1)	0	218 (72.9)	0	71 (23.7)	0	113 (37.8)
1	1 (0.3)	1	17 (5.7)	1	24 (8.1)	1	32 (10.7)	1	115 (38.5)	1	93 (31.1)	1	54 (18.1)	1	121 (40.5)	1	88 (29.4)
2	2 (0.7)	2	11 (3.7)	2	5 (1.7)	2	14 (4.8)	2	57 (19.1)	2	58 (19.4)	2	21 (7.0)	2	55 (18.4)	2	63 (21.1)
		3	4 (1.4)	3	6 (2.0)	3	5 (1.7)	3	24 (8.0)	3	48 (16.0)	3	6 (2.0)	3	52 (17.4)	3	30 (10.0)
		4	9 (3.0)	4	3 (1.0)	4	8 (2.7)	4	7 (2.3)	4	34 (11.4)					4	5 (1.7)
		5	2 (0.7)	5	2 (0.7)	5	4 (1.4)										
		6	3 (1.0)	6	3 (1.0)	6	3 (1.0)										
		7	3 (1.0)	7	2 (0.7)	7	1 (0.3)										
		10	1 (0.3)	8	3 (1.0)	8	3 (1.0)										
		25	1 (0.3)	11	1 (0.3)	9	2 (0.7)										
				12	2 (0.7)	10	1 (0.3)										
				14	1 (0.3)	11	3 (1.0)										
				23	1 (0.3)	13	1 (0.3)										
				25	1 (0.3)	14	1 (0.3)										
				26	1 (0.3)	19	2 (0.7)										
				62	1 (0.3)	20	1 (0.3)										
						21	2 (0.7)										
						26	1 (0.3)										
						31	1 (0.3)										
						34	1 (0.3)										
						36	1 (0.3)										
						46	1 (0.3)										
						67	1 (0.3)										

Abbreviations: BG-PVS, basal ganglia perivascular space; CMB, cerebral microbleed; CSO-PVS, centrum semiovale perivascular space; cSS, cortical superficial siderosis; DWMH, deep white matter hyperintensity; PVWMH, periventricular white matter hyperintensity; SVD, small vessel disease.
